# Evaluation of the Effect of Atenolol Induced Depression in Cardiac Output on Its Own Excretion through Urine Analysis

**Published:** 2019

**Authors:** Safeer Khan, Wajahat Mahmood

**Affiliations:** *Department of Pharmacy, Comsats Institute of Information Technology (CIIT) Abbottabad, Khyber Pakhtun Khwa, Pakistan.*

**Keywords:** Cardiac Output, Atenolol, Urine Analysis, Excretion, Half-life

## Abstract

The role of Pharmacist in making the therapeutic decisions for safe and effective therapy is increasing all over the world. However, there are many aspects of drugs in making these decisions that are less commonly studied such as the correlation of cardiac output with pharmacokinetics of drugs. The cardiac output, besides the other factors, is also affected by drugs like atenolol. Therefore, the objective of the present open labeled study was to know the effect of reduced cardiac output induced by atenolol on its own excretion parameters. After taking the informed consent, five healthy volunteers were selected for the study. Atenolol tablet at a dose of 50 mg, 75 mg and 100 mg for three consecutive days were given to all the volunteers. The echocardiography and renal function clinical tests were conducted prior and 5 h after dosing and the urine samples were collected at 5 and 10 h post dosing. The prepared samples were analyzed for atenolol by High-Performance Liquid chromatography. For comparison of atenolol excretion for three days, One-way repeated measure Analysis of Variance statistical test was used as Wilks’ Lambda = 0.2, F (2, 3) = 5.986, *p* < 0.1, multivariate partial squared = 0.8. These results showed that atenolol affects its own pharmacokinetics by prolonging its excretion half-life.

## Introduction

The role of pharmacist in the therapeutic decisions for safe and effective therapy is increasing every day. For this purpose, the pharmacist should be well aware of every aspects of the drugs. But there are many examples of well*-*known drugs, for which clinical aspects are not characterized to enable the pharmacist to practice them in the health care settings. One such example is the atenolol, where the correlation between the administered dose and its subsequent effect on its own excretion and other co**-**administered drugs is not studied in an effective manner. 

Cardiac output (CO) can be defined as the amount of blood in liters pumped by the heart in unit time of one min (1). On average, a healthy adult's heart pumps 5 L every min that also depends on some physiological and non-physiological factors (2). The drugs that affect the CO may have a positive or negative effect (3). 

One of the examples of drugs that affect the CO negatively is Beta blockers. The effect of Beta blockers to decrease the CO is attributed to their blockage of the β1 receptor in the heart (4). 

Atenolol is a hydrophilic cardioselective Beta blocker. At 100 mg of dose it prevents 80% of β1 actions and 20% of β2 actions (5). After oral administration, the drug is absorbed but in an incomplete fashion and is well absorbed at pH 7.5. Due to its hydrophilic nature, only 40-50% of the drug reaches the systemic circulation (6). Atenolol has only less than 10% of metabolism by the liver. About 85-95% of the drug is excreted unchanged in urine that can be completed in 7 to 9 h (7). Although plasma half**-**life is 6**-**8 h, it is increased in renal failure. Due to these reasons urine is the ideal sample for analysis of atenolol after its excretion (6). 

There are many factors which affect the pharmacokinetics of the drugs (8). Out of these factors, cardiac output is less commonly studied to know its correlation with Pharmacokinetics of the drugs (9). 

When the heart is functioning normal (CO=4**-**8 L/min), our kidney receives 19% in adult male and 17% in an adult female of the cardiac output (10). But there are many conditions in which cardiac output is impaired (9). When cardiac output is impaired then it decompensates the perfusion of the drugs to the organs like kidneys and liver (11). This response can affect the pharmacokinetics parameters of the drugs especially excretion and metabolism (8). 

Our present study is designed mainly to analyze the urine sample for atenolol to answer the following question “Does the decrease in cardiac output induced by atenolol therapy affect the half**-**life of atenolol and all those drugs which use kidneys as the main excretory route when co-administered with atenolol?”

## Experimental

*Ethical Consideration*

All experimental interventions on study subjects were performed in accordance with the guidelines, ethical principles, and regulations approved by the Research Ethics Committee (REC), Department of Pharmacy, COMSATs Institute of Information Technology (CIIT), Abbottabad, Pakistan.

*Subject Selection*

Five healthy volunteers: one female and four male who have no medical condition and surgical disease were included in the study. The mean age was 28.2 years (R = 18 to 49) while the mean body surface area (BSA) was 24.4 with a maximum of 30.2 and minimum of 21.3 kg/m^2^. All the volunteers in our study were non**-**smokers. The samples collected were given a specific code for the purpose of identification and for confidentiality matters. All the individuals selected had normal cardiac and renal functions that were confirmed by the clinical test of echocardiography and renal function test before intimation of the study.

**Table 1:** Clinical assessment results

**Subject Code**	**Day**	**Serum Creatinine (mmol/L)**	**Serum Urea (mmol/L)**	**Heart Rate/ min**	**LVEF (%)**	**Cardiac Output (mL/min)**
	01	70	2	80	70	5600
12	02	75	2.5	83	72	5976
	03	40	5.2	75	66	4950
Mean		61.7	3.2	79.3	69.3	5508.7
	01	75	2.2	75	68	5100
22	02	68	2.6	70	65	4550
	03	71	5.5	73	62	4526
Mean		71.3	3.43	72.7	65	4725.3
	01	56	4.1	72	76	5472
32	02	138	6.6	68	70	4760
	03	131	6.3	69	68	4692
Mean		108.3	71.3	69.7	71.3	4974.7
	01	90	2.1	78	68	5304
42	02	83	3.1	72	65	4680
	03	97	2. 6	73	63	4599
Mean		90	2. 6	74.3	65.3	4861
	01	92	3.2	56	69	4692
52	02	195	4.4	56	65	4550
	03	158	6	56	64	4352
Mean		148.3	4.5	56	66	4531.3

**Table 2:** Urine excretion of atenolol

**Subject Code**	**Day**	**Dose administered**	**Atenolol excretion**	**Total atenolol excretion (mg)**		**% atenolol excretion over**	**Rate of atenolol excretion**
		**(mg)**	**(µg/mL)**	**5 (h) 510 (h)**		**10 (h)**	**(mg/h)**
	1	50	135.7	4.9	16.2	42.2	4.2
12	2	75	74	3.8	21	33	4.9
	3	100	99.2	3.1	10.4	31.5	6.3
Mean			102.9	3.9	15.8	35.5	5.1
	1	50	253	9.2	18	42.2	5.4
22	2	75	77	14.4	17.2	47.2	6.4
	3	100	607.6	11	41	25	10.4
Mean			312. 5	11.5	25.4	38.1	7.4
	1	50	46.5	8.9	3.5	23	2.3
32	2	75	9.3	2.7	0.5	4.4	0.6
	3	100	13.6	0.6	3.6	4.2	0.8
Mean			23.2	4	2.6	10.4	1.3
	1	50	24.9	10.9	3.2	28	2.8
42	2	75	2.8	0.6	0.7	1.7	0.3
	3	100	16.2	0.3	2.8	3.2	0.6
Mean			14. 6	3.9	2.2	10.9	1.2
	1	50	2.9	0.03	1.5	3.1	0.3
52	2	75	5.3	0.5	0.9	1.9	0.3
	3	100	1.5	0.2	0.4	0.6	0.11
Mean			3.2	0.3	0.9	1.9	0.2

**Figure 1:**
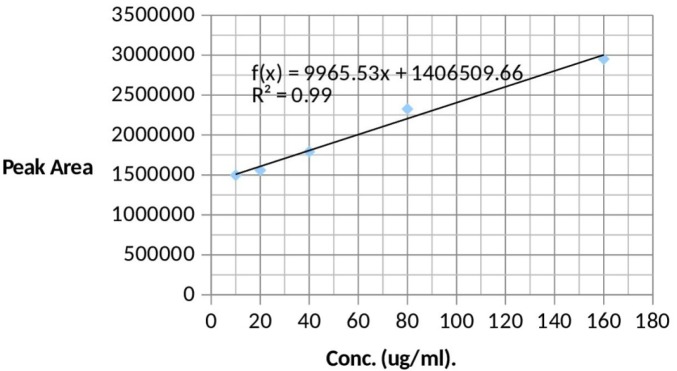
Calibration curve for atenolol. Peak area = 9956 concentration (µg/mL) + 106. An R2 of 0.986 indicates that the regression line fits our data

**Figure 2:**
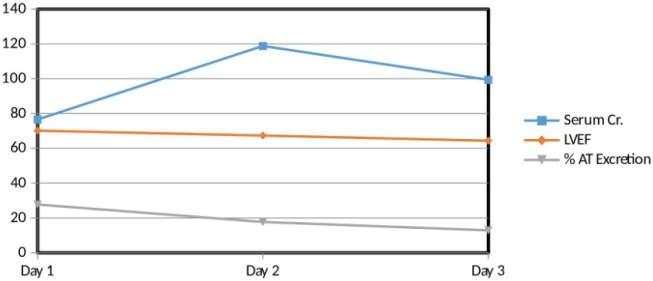
Interrelation between serum creatinine, left ventricular ejection fraction (LVEF) and percent atenolol excretion. There is a direct relation between LVEF and percent AT excretion. On the other hand, there is an inverse relation of serum Creatinine with LVEF as well as with percent AT excretion

**Figure 3:**
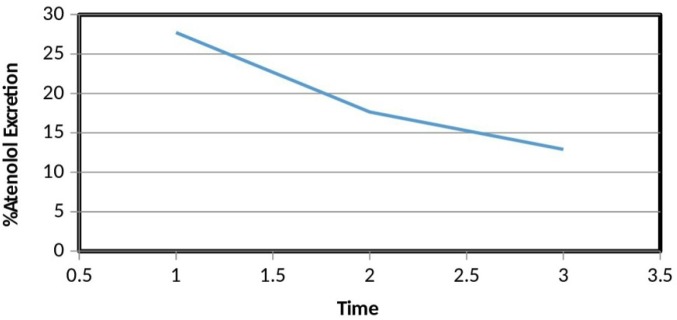
Percent atenolol excretion versus time. Percent atenolol excretion of our subjects over three days dosing significantly decreased over time

*Study Protocol*

The study was performed according to an open*-*labeled design. All the subjects were instructed not to take any type of medication or tonics before seven days of intimation of drug administration. 

Atenolol as tablet form at a dose of 50 mg, 75 mg and 100 mg was given orally with 250 mL of water to all the volunteers in three consecutive days respectively. After 5 h of dose administration, 3 mL of blood sample was collected in a plain red top tube with a 5 mL plastic syringe on each day of dosing for the purpose of investigation of renal functions. To investigate the cardiac functions, the echocardiography test was performed at 5 h post dosing on each day of the three days. All urine voided were collected from each subject at a frequency of 5 h and 10 h post dosing respectively on each day so that a total of 6 samples were collected from each subject. Continuous monitoring of the heart rate and blood pressure was also done for all the individuals up to the 10 h post dosing. The echocardiography, heart rate, and blood pressure tests were done following 15 min of rest.

The Cardiac output (CO) was determined by the method of transoesophageal echocardiography technique. Both echocardiography and heart rate tests were performed at the same time. To avoid large fluctuation by the physiological and non**-**physiological factors, the final heart rate used for the measurement of CO was taken as the mean of three heart rate values determined at three different times with a gap of 5 min in between. 

The volume of all urine samples were noted in calibrated graduated cylinder and 30 mL of each sample was stored in a 100 mL dry sterile urine bottle in the refrigerator until they were used.

*Sample Preparation*

The 20 mL urine was taken from the urine sample in a dry sterile 100 mL urine bottle. The samples were then centrifuged for 5 min at 1000×g. The supernatant transparent layer from each sample was collected in 50 mL round bottom flask to which 2 × 5 mL portions of methanol (HPLC grade) were mixed. To facilitate dissolution, the mixture was shaken on a magnetic stirrer for 5 min. With the help of precision pipette, a 5 mL sample was taken from the upper portion of the sample and mixed with 5 mL of the mobile phase. The final 10 mL sample was then stored in the refrigerator until analysis. 

Pooled normal human blank urine was spiked by the use of precision pipette with appropriate volumes of standards (10 to 160 ug/mL) to achieve standard solutions containing 10 ug/mL, 20 ug/mL, 40 ug/mL, 80 ug/mL and 160 ug/mL. These solutions were used to obtain a standard curve (Figure 1). 

*Sample Analysis*

The HPLC system used was of Perkin Elmer series 200 and the mobile phase used was a mixture of 50 mM Phosphate buffer prepared in HPLC grade water and HPLC grade Acetonitrile in a ratio of 50:50 v/v adjusted at a pH 7.

*Statistical Analysis*

All statistical calculations were performed with the Statistical Product and Service Solutions (SPSS) for Windows, version 19.0.

To detect the effect on the rate of excretion over three days, three groups were named as day 1, day 2, and day 3. Their percent rate of excretion was compared using repeated measure Analysis of Variance (ANOVA) statistical test. The significance value of Wilks lambda was used to check the significance of percent atenolol excretion in the three consecutive days. An ANOVA *P*-value of 0.1 was considered as statistical significance. Partial eta squared was used to check the effect size. Pair**-**wise comparison was also used to compare the % atenolol excretion between day 1 and day 2, day 1 and day 3 and day 2 and day 3 respectively. 

## Results

Five subjects were entered into the study, and they all completed it without any incident.

*Clinical Assessment Results*

There was 11%, 13%, 12%, and 3% decrease in CO on the 2^nd^ day of four subjects. The results of CO of these four subjects on 3^rd^ day showed 1%, 1%, 2%, and 4% decrease in CO respectively. The CO of one of the subject show results which were quite different from the other subjects *i.e.* 7% increase on the 2^nd^ day and 17% decrease on the 3^rd^ day. 

In three out of five subjects, the serum creatinine increased on the 2^nd^ day and then decrease down on the 3^rd^ day. The serum creatinine becomes abnormal on the 2^nd^ day and 3^rd^ day in two of the subjects. In the case of serum urea, all the observed values were normal and did not significantly change (Table 1). 

*Urine excretion of atenolol*

According to the results subject code, 22 has the highest mean rate of atenolol excretion *i.e.* 7.41 mg/h as well as highest mean percent atenolol excretion *i.e.* 38.1%. On the other hand, subject code 52 has the lowest mean rate of excretion *i.e.* 0.23 mg/h as well lowest mean percent atenolol excretion *i.e.* 1.9% when we administered a 50 mg dose on the 1^st^ day, 75 mg on the 2^nd ^day and 100 mg dose of atenolol on the 3^rd^ day (Table 2). 

*Interrelation between Serum Creatinine, Left Ventricular Ejection Fraction (LVEF) and Percent Atenolol excretion*

In general, there was a direct relation between LVEF and percent atenolol excretion. On the other hand, there was an inverse relation of serum creatinine with LVEF as well as with percent atenolol excretion. But here is an exception of subject code 12. The 3^rd^ day results of subject code 12 show a slight direct relation between serum creatinine with LVEF as well as with percent atenolol excretion (Figure 2).

## Discussion

The Cardiac Output (CO) is one of the main factors involved in the maintenance of metabolism of the body. Low Cardiac Output (CO) impairs the body metabolism and thus all the associated functions (12). 

The reduction in CO shown by our results are in agreement with many of other studies. Riesen *et al*., 2011 conducted a study on ten healthy cats to compare the effect of Ivabradine and atenolol on cardiac rate and echocardiographic variables. After administration of atenolol for four weeks, the heart rate was decreased from the baseline 247/min to 165/min and Ejection fraction decreased from the baseline 67% to 61% for atenolol (13). Dhakam *et al*., 2008 administered 50 mg of atenolol and placebo for five weeks to sixteen hypertensive patients never been treated. They found that the heart rates were to be 80 ± 3 for the placebo and 57 ± 1 for atenolol treated patients (14). 

We found that CO decrease earlier and then increased on the 3^rd^ day. The work of Galcea-Tomas *et al*., 2001 supports our results who concluded that atenolol causes an early significant reduction of heart rate and CO (15). The extraordinary results of subject 12 in our study *i.e.* 7% increase on the 2^nd^ day and 17% decrease on the 3^rd^ day can be attributed to non-physiological factors which affect the CO (3). 

In both the disease and normal conditions, the glomerular filtration rate (GFR) is considered as the most appropriate measure for kidney functions (16). Serum creatinine is used as an alternative for estimation of GFR as it is easy to measure then to calculate GFR. An exponential relation exists between serum creatinine and GFR (17). Similarly, serum creatinine is typically used as a marker for acute changes in the functions of kidneys (18). 

The mechanism by which the atenolol affects the kidneys is through an extrinsic renal blockage (19). A study conducted by Bakris *et al*., 1997 supports our results. They compared the atenolol versus verapamil with respect to the effect on renal functions and concluded that there was a 50% or more increase in serum creatinine in patients taking atenolol as compared to those patients who are on verapamil therapy *i.e.* 32 ± 9% versus 16 ± 7% at *P* < 0.05 (20). Atenolol affects the functions of the kidneys and has more effect on protein excretion (21).

If we relate our results of serum creatinine to CO of each subject, it shows that the decrease in renal functions was secondary to the decrease in CO that was caused by administration of atenolol. Our conclusion is supported by the study of Ochiai *et al*., 2011 who showed that low CO is one of the important factors for worsening the functions of the kidneys (22). 

The statistical analysis concludes that a significant difference was present between the percent atenolol excretion of the same subjects over three days as Wilks’ Lambda = 0.2, F (2, 3) = 5.986, *P* < 0.1, multivariate partial squared = 0.8. The results suggest that % atenolol excretion of our subjects over three days dosing significantly decreased over time. According to Cohen, multivariate partial squared of 0.14 or above shows a large effect (23). In this respect, the effect of atenolol on its own excretion half**-**life in our study was large as multivariate partial squared = 0.8. 

We have determined the concentration of atenolol in 10 h post dosing sample. In these 10 h post dosing sample, the concentration of atenolol over three days was decreasing over the days. It means that the time to excrete atenolol from the body by the kidneys has increased. In other words, the excretion half**-**life of atenolol prolonged over the time (Figure 3).

In summary, atenolol decreases the CO that lowers the renal blood flow and in turn the reduced renal blood flow prolongs the excretion half**-**life of atenolol. Our results are in agreement with the study that was conducted on old aged people. In older people there is a decrease in heart rate with an increase in the peripheral resistance. These conditions are also associated with the increased level of plasma serum creatinine. Therefore, in an older age, the excretion and pharmacokinetics of some drugs are affected like digoxin whose excretion decreases with age. Similarly, the prolong half**-**life of intravenous furosemide in elder subjects is due to reduce renal flow secondary to decreased CO. In older subjects the same mechanism happens with most of the ACEIs which are excreted through renal route (11). Most of the barbiturates, Morphine and Fentanyl used in anesthesia decreases blood flow towards kidneys and thus lowers GFR. This lowered GFR is secondary to reduced CO that is caused by these drugs. The lower GFR intern decreases the rate of urine formation (24). We can also support our results by taking the example of dopamine which increases the CO by acting on dopaminergic receptors. This leads to increase blood flow to the kidneys and results in natriuresis. Due to this reason, it is used as a most common drug for low CO with oliguria (25). 

## Conclusion

The administration of atenolol affects its own pharmacokinetics by prolonging its excretion half**-**life. This effect is secondary to the lower blood perfusion towards the renal system which is caused by reduced cardiac output induced by atenolol. 
